# Transcriptome analysis of classical blood cells reveals downregulation of pro-inflammatory genes in the classical monocytes of long COVID patients

**DOI:** 10.3389/fimmu.2025.1710783

**Published:** 2025-11-07

**Authors:** Florian Fricke, Franz Mai, Christine Wossidlo, Felix Steinbeck, Wendy Bergmann-Ewert, Marcel Kordt, Karin Kraft, Britta Müller, Emil C. Reisinger, Brigitte Müller-Hilke

**Affiliations:** 1 Core Facility for Cell Sorting and Cell Analysis, University Medical Center Rostock, Rostock, Germany; 2 Department of Tropical Medicine and Infectious Diseases,University of Rostock, Rostock, Germany; 3 Institute for Immunology, University Medical Center Rostock, Rostock, Germany; 4 Chair of Naturopathy, University Medicine Rostock, Rostock, Germany; 5 Institute of Medical Psychology and Medical Sociology, Rostock University Medical Centre, Rostock, Germany

**Keywords:** immune landscape, immune tolerance, monocytes, SARS-CoV-2, long COVID, scRNAseq

## Abstract

**Introduction:**

Despite extensive research, the pathogenesis and predispositions underlying long COVID (long-term coronavirus disease 2019) remain poorly understood.

**Methods:**

To address this, we analyzed the immunological landscapes of 44 patients with long COVID and 44 matched convalescents using single-cell RNA sequencing (scRNA-seq) of peripheral blood mononuclear cells (PBMCs) and validated the findings with plasma cytokine measurements via Luminex technology.

**Results:**

While the immune cell compositions showed minimal quantitative differences only among natural killer (NK) cells, the transcriptome analyses identified distinct gene expression patterns, particularly in classical monocytes: patients with long COVID exhibited downregulation of the inflammation-associated genes, including *IL1B* and *CXCL2*. Imputation of the transcription factor activity hinted at a reduced inflammasome activity (via *SNAI1*) and an impaired monocyte differentiation (via *ATF2*) in long COVID. The RNA velocity data supported the presence of immature classical monocytes in these patients.

**Discussion:**

These findings show that monocytes might be dysregulated and/or exhausted in patients with long COVID.

## Introduction

Since its emergence in late 2019, severe acute respiratory syndrome coronavirus 2 (SARS-CoV-2) has caused a global pandemic resulting in millions of deaths (1) and has posed significant public health challenges. While accelerated vaccine development and the evolution of viral variants have led to a reduction in severe acute coronavirus disease 2019 (COVID-19) cases (2), another clinical entity, i.e., "long COVID", has gained attention. Long COVID describes the persistence of symptoms lasting for at least 4 weeks after infection that cannot be explained by another diagnosis (3). It is estimated that approximately 10% of individuals infected with SARS-CoV-2 will experience long COVID, although the data vary between publications (4). Since the term "long COVID" has become established in the language used by patients and also in science, we decided to use it in our study instead of the term "post-COVID", as defined by the World Health Organization for a condition that specifies symptom onset within three months after SARS-CoV-2 infection and persistence for at least two months (5). The characteristic symptoms include fatigue, shortness of breath, and cognitive impairment; however, over 200 symptoms have been described in patients with this condition (6). The diverse clinical phenotypes of patients with this condition hamper diagnosis and adequate treatment and call for a deeper understanding of its pathogenesis.

The risk factors for the development of long COVID include female sex, age, abnormal BMI, and severe previous courses of SARS-CoV-2 infection (7–9). However, it remains unclear whether and to what extent genetics predispose and which mechanisms contribute to this condition (10). Hypotheses to explain the latter include the persistence of a viral reservoir, potentially within the gastrointestinal tract (11–13), and the reactivation of latent viruses, such as Epstein–Barr virus (14–16). Others have focused their attention on the impact of SARS-CoV-2 on the vascular system (17–19). Our own focus is on the immunological landscape, as both autoimmunity (20–22) and a perturbation of the peripheral immune system (23–25) have been discussed as potential underlying pathology. Along these lines, previous single-cell RNA sequencing (scRNA-seq) studies have demonstrated distinct immunological subsets in patients with long COVID, including increased myeloid lineage cells with downregulated immune pathways in two cases (26). Another study on 69 infected individuals, including 21 with long COVID, reported significant perturbations in gene expression until at least 6 months post-infection, with long COVID patients failing to revert to their pre-infection state (27). In contrast, another independent study on 10 patients showed that the majority of the immunological changes in long COVID resolved 24 months after infection (28).

Despite significant global research efforts, the underlying mechanisms of long COVID remain incompletely understood. To further the current knowledge, we performed scRNA-seq on the peripheral blood mononuclear cells (PBMCs) of 44 patients with long COVID and 44 matched convalescents in combination with plasma protein profiling in order to gain detailed insights into the cellular and molecular changes associated with long COVID.

## Methods

### Recruitment and characterization of the study cohorts

**Figure 1:**
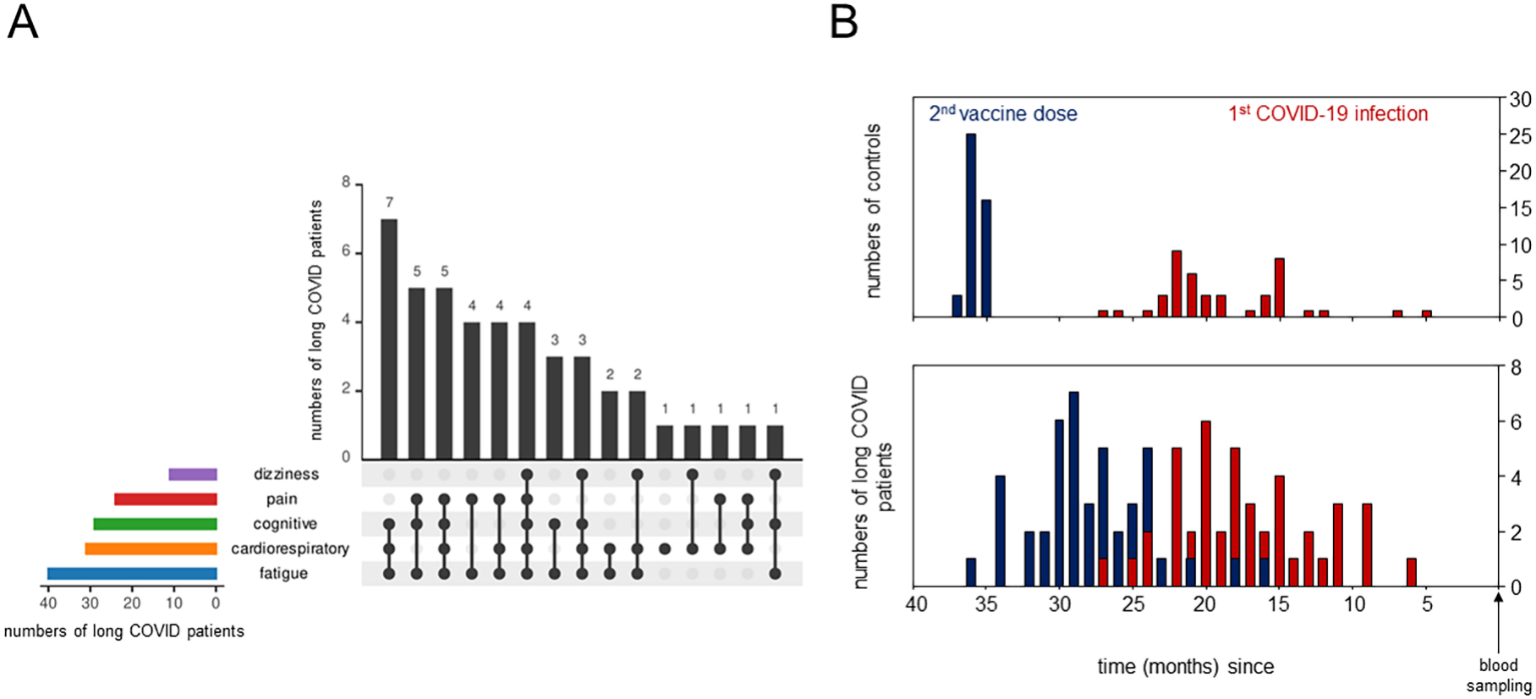
Detailed description of long COVID patients and the control cohorts. **(A)** UpSet plot summarizing the pooled patient groups, with *horizontal bars* representing the number of patients in each symptom group and *vertical black bars* representing the symptom combination groups. **(B)** Bar plots showing the time (in months) between blood sampling and the second vaccine dose in *blue* and between blood sampling and the primary severe acute respiratory syndrome coronavirus 2 (SARS-CoV-2) infection in *red*.

A total of 44 patients with long COVID were recruited from the outpatient clinic of Rostock University Medical Center. Selection was based on the order of their visit, and diagnosis followed the WHO definition of “Post COVID-19 condition (long COVID)” (5, 29). The symptoms, disease progression, comorbidities, previous treatments, and quality of life are summarized in **Figure 1A**. Health-related quality of life (HRQoL) was assessed using the Short-Form 12 (SF-12) (30) and the Bell Disability Score (31). Routine laboratory tests included complete blood counts, coagulation parameters, C-reactive protein (CRP), liver enzymes, renal parameters, vitamin B12, folate, 25-OH-vitamin D, and thyroid-stimulating hormone (TSH), as well as basic autoimmune diagnostics. Differential diagnoses were excluded by the consulting cardiologists and pneumologists. A total of 44 age- and sex-matched controls were recruited from a monitored healthcare worker cohort (32–34). Selection was based on primary messenger RNA (mRNA) vaccination and on optimal matching with patients. Of the controls, 43 were convalescent from SARS-CoV-2 infection and one was uninfected.

### Ethics commitment

This study was approved by the Ethics Committee of the Rostock University Medical Center under file no. A 2020-0086 and no. A 2023-0081. Written informed consent was provided by all participants.

### PBMC isolation

Venous blood was collected into EDTA vacutainers. PBMCs were isolated using SepMates (StemCell, Vancouver, BC, Canada) with Ficoll-Paque PLUS (Cytiva, Marlborough, MA, USA). Aliquots of 1–3 × 10^6^ cells each were stored at −80°C until further processing.

### Single-cell capture and RNA sequencing

After thawing, 180,000 PBMCs were labeled with BD Human Multiplexing Sample Tags (Becton Dickinson, Franklin Lakes, NJ, USA) for the multiplexing of 12 samples per run. The viability of PBMCs was assessed via staining with Calcein and DRAQ7 (Thermo Fisher Scientific, Waltham, MA, USA). RNA was harvested using the Single-Cell Capture and cDNA Synthesis protocol with the BD Rhapsody Single-Cell Analysis System, and a total of eight runs were performed to enable single-cell capture from all patients and controls. Each run contained a balanced mixture of patient and control samples plus a spike control (isolated CD3^+^ T cells from an independent donor) to monitor potential batch effects. A total of 40,000–60,000 cells were pooled and loaded onto the Rhapsody cartridge. The incubation times for settlement of the cells varied between 15 and 25 min. The RNA libraries for scRNA-seq were prepared using the Library Preparation Protocol for mRNA Whole Transcriptome Analysis and Sample Tag according to the manufacturer’s instructions and were purified using AMPure beads (Beckman Coulter, Brea, CA, USA). The libraries were indexed using a run-specific primer and were quantified and quality-checked using a Qubit 3.0 Fluorometer (Thermo Fisher Scientific, Waltham, MA, USA) and an Agilent 4200 TapeStation System with the Agilent HS D5000 screen tape assay (Agilent Technologies, Santa Clara, CA, USA). Sequencing was performed by Novogene Europe (Cambridge, UK, Munich subsidiary) using Illumina technology.

### scRNA-seq data analysis and quality control

Raw scRNA-seq reads were aligned to the human genome and annotated by cell type using the BD Rhapsody™ Sequence Analysis Pipeline 2.2.1, complemented by manual assignment as previously described in Hillman et al. (35). Subsequent analysis was conducted using the Seurat 5.1.0 package in R, version 4.4.1 (R Core Team 2024, Vienna, Austria) (36, 37).

Quality control procedures involved the exclusion of cells exhibiting either an excessive or an insufficient number of unique RNA products or more than 25% of mitochondrial genes. A total of 132,230 PBMCs could successfully be recovered (44 patients and 44 controls). Of these, 118,843 passed quality control. Cartridge batch effects were assessed using a cell mix score calculated using the CellMixS 1.7.1 package for R for the CD3^+^ spike cells, the aim of which was to statistically validate the visual impression of homogeneous distribution provided by the UMAP (uniform manifold approximation and projection). This score is based on the calculation of a *p*-value for each cell, which represents the probability of belonging to a common underlying population (38). Subsequent analysis involved the normalization and scaling of the reads.

### Cytokine profiling

Cytokines were measured using a custom-designed Luminex^®^ Discovery Assay panel (R&D Systems, Minneapolis, MN, USA), with selection based on the identified differentially expressed genes (DEGs). The assay was performed according to the manufacturer’s protocol using undiluted plasma samples. Data acquisition was carried out using the Luminex^®^ 100/200™ System, with calculations performed using xPONENT^®^ 3.1 software. The lowest manufacturer standard was considered as the lower limit of detection (LLOD). No extrapolation was performed.

### Measurement of IgG antibodies against the SARS-CoV2 nucleocapsid

Cryopreserved plasma samples from both patients and controls were analyzed using an enzyme-linked immunosorbent assay (ELISA) specific for immunoglobulin G (IgG) antibodies against the nucleocapsid protein of SARS-CoV-2 [anti-SARS-CoV-2-NCP-ELISA (IgG)] (EUROIMMUN Medizinische Labordiagnostika AG, Lübeck, Germany) following the manufacturer’s protocol. The samples were centrifuged at 10,000 × *g* for 5 min prior to 101-fold dilution. Photometric measurements were obtained at 450 nm with a reference wavelength of 620–650 nm using the Infinite M200 spectrophotometer (Tecan, Männeheim, Switzerland). The results were calculated as the ratio of the sample extinction to that of the calibrator.

### Statistics

DEGs were determined using a non-parametric Wilcoxon rank-sum test and were classified based on a fold change greater than 3 and a (Bonferroni-corrected) *p*-value less than 10^−18^. UMAPs were constructed using nearest-neighbor clustering of all the genes expressed. Spearman’s rank correlation coefficient was employed for correlation analyses. Heatmaps were constructed using a column/row-wise clustering approach with complete linkage and Euclidean distance metrics. Gaussian distribution was assessed using Kolmogorov–Smirnov tests. The Mann–Whitney *U* test was employed for the comparison of data that did not adhere to a normal distribution. Statistical analysis was performed using SPSS version 29.0.1.1 (IBM, Armonk, NY, USA) and GraphPad Instat version 3.1 (GraphPad Software, Boston, MA, USA).

### Transcription factor activity

Estimation of transcription factor (TF) activity was performed using the DoRothEA R package version 1.16.0, a gene regulatory network comprising signed interactions between TFs and their target genes (39–41). The VIPER algorithm (42) was employed to estimate the activity of the TFs from the expression data of all monocytes of all genes. Group comparisons were made based on the mean differences.

### RNA velocity

The raw reads of all genes were divided into spliced and unspliced read matrices before estimation of the RNA velocities using gene-relative slopes. The velocity graph was visualized on the previously calculated UMAP of all monocytes embedding, employing a correlation-based transition probability matrix within the *k*-nearest neighbor graph. All analyses were performed using velocyto.R and velocyto.py (43).

### Pathway analysis

Pathway analysis was conducted with ShinyGO software (44), which compared all downregulated genes in all monocytes of the long COVID cohort with the Reactome 2022 (45) and the Kyoto Encyclopedia of Genes and Genomes (KEGG) 2021 (46–48) databases. Gene set enrichment analyses (GSEA) were performed by alignment of the normalized counts of all monocytes with the Hallmark Gene Sets of Molecular Signatures Database (49–51). Enriched pathways were identified using the normalized enrichment score (NES) and the false discovery rate (FDR).

## Results

### Peripheral immune cell compositions in long COVID patients were inconspicuous

We analyzed the PBMCs from 44 individuals diagnosed with long COVID using scRNA-seq, with the detailed clinical characteristics, comorbidities, and treatments summarized in **Table 1**. Majority of the patients reported mild (*n* = 27) or moderate (*n* = 13) initial SARS-CoV2 infections (29). Quality of life, assessed in 21 and 16 long COVID patients using the Short-Form 12 (30) and the Bell score (31), respectively, was significantly reduced compared with that of standardized populations [MCS (mental component summary): *t (*20) = −13.17, *p* < 0.001; PCS (physical component summary): *t (*20) = −15.33, *p* < 0.001]. Patients were grouped according to symptoms such as dizziness, pain, cognitive or cardiorespiratory constraints, and fatigue. The number of patients in each symptom combination group is presented in **Figure 1A**. The severity of the symptoms and post-exertional malaise, classified as extreme exhaustion after minimal stress, were mentioned, when available. Controls (*n* = 44) were age- and sex-matched, mostly convalescent (*n* = 43) plus one naive to SARS-CoV-2 (*n* = 1), as determined based on the anti-nucleocapsid antibodies. Both groups received comparable numbers of SARS-CoV2 vaccinations and had received mRNA vaccines only (**Table 1**). The controls, mainly healthcare workers, were primed and boosted at the turnaround 2020/2021, followed by the patients in the spring/summer of 2021. First SARS-CoV-2 infections occurred mainly in 2022, suggesting Omicron as the predominant variant in all study participants (52). **Figure 1B** summarizes, for both cohorts, the time intervals between blood sampling and completion of the primary immunization, as well as between blood sampling and first infections.

**Table 1:** Demographics, vaccinations, and infections of the study cohorts.

Parameter	Patients (*n* = 44)	Controls (*n* = 44)	*p*-value
Men/women, *n*	9/35	9/35	1.0^b^
Age (years), median (range)	54 (19–79)	51 (26–63)	0.5202^c^
No. of vaccinations, median (min–max)	3 (3–6)	3 (3–5)	0.1055^c^
Anti-nucleocapsid antibodies, median (min–max)^a^	0.6811 (0.2490–8.414)	2.479 (0.3222–7.898)	0.0086^c^

a Ratios that are a relative measure for the concentration of antibodies in plasma.

b Fisher’s exact test.

c Mann–Whitney *U* test.

At the time of blood collection between July 2023 and February 2024, the controls, as part of a vaccination study (32), had been regularly assessed for anti-nucleocapsid antibodies, revealing a median of two infections each. Regarding the patients, the infection that triggered long COVID had been documented; thereafter, any reliable information on additional SARS-CoV-2 infections was sketchy. We therefore quantified the patients’ anti-nucleocapsid IgG antibodies at the time of blood collection and found evidence of recent infections in some patients, but also confirmed fewer and/or longer ago infections for the majority, resulting in a significantly lower median for the anti-nucleocapsid titers in patients compared with the controls (**Table 1**).

Quality control of the scRNA-seq included the exclusion of cells with aberrant counts of unique RNA products (<200 and >4,000) and cells expressing more than 25% of mitochondrial genes. Assessment of batch effects and calculation of the cell mixing scores of the spike controls from each experimental run confirmed a high degree of comparability between all experimental runs, justifying the combined processing of all data (**Supplementary Figures S1D–F**).

**Figure 2:**
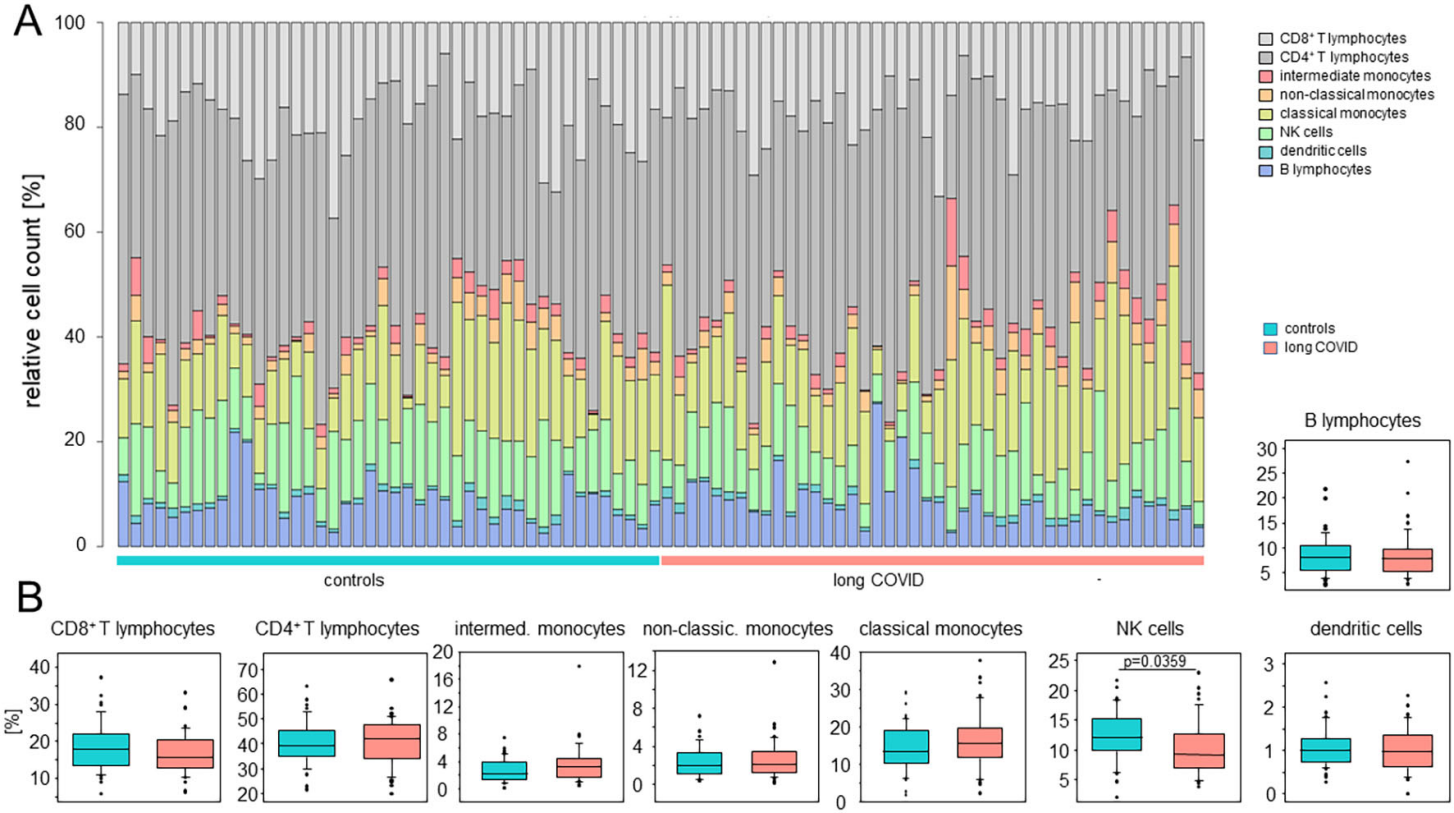
The peripheral immune cell compositions in long COVID patients were inconspicuous. **(A)** Stacked bar plots indicating the relative cell counts in each sample. Cell types, long COVID patients, and controls are color-coded, as shown on the *right*. **(B)** Box plots summarizing the percentages determined for the controls and the patients for each cell type. *Boxes* represent 25, 50, and 75 percentiles, and *lower* and *upper whiskers* indicate 10 and 90 percentiles, respectively. Outliers are shown. The *p*-values were determined using two-sided Mann–Whitney *U* test. No adjustments for multiple comparisons were made.

Using the BD Rhapsody™ Sequence Analysis Pipeline on the basis of the highly expressed genes, in conjunction with a manual assignment of monocytes according to the expression of CD14 and CD16, as previously described in Hillman et al. (35), allowed for the identification of eight distinct cell types (**Figure 2A**). In order to mitigate potential biases due to sample-to-sample variations in cell yields, we here compared the relative cell counts. **Figure 2A** shows comparable immune cell compositions between patients and controls, with only minor differences in NK cells (**Figure 2B**).

### Classical monocytes of long COVID patients display significant alterations in their immune signatures

**Figure 3:**
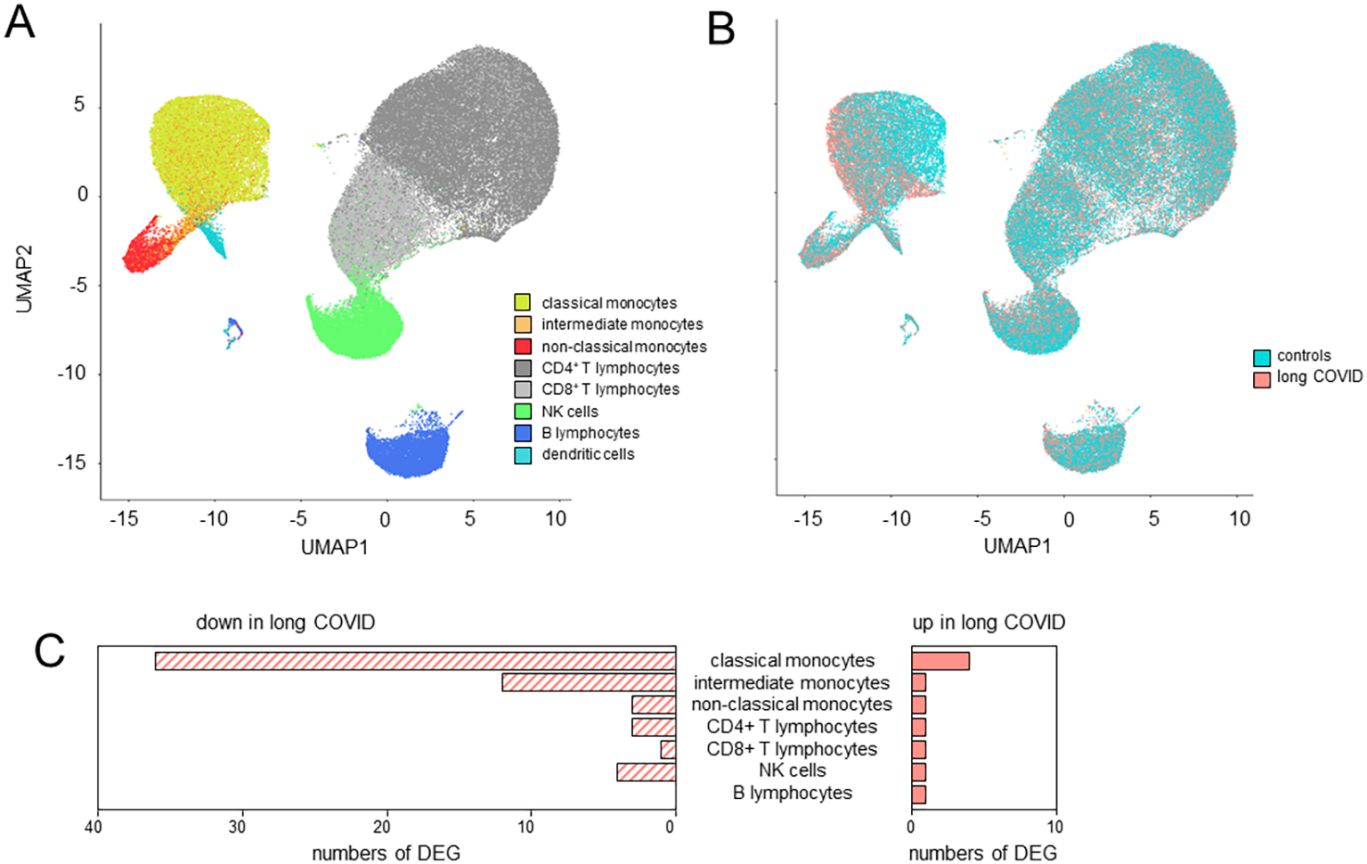
Classical monocytes in long COVID patients showed significant alterations in their immune signatures. **(A)** Uniform manifold approximation and projection (UMAP) of the single-cell RNA sequencing (scRNA-seq) data identified eight distinct cell types as defined by the BD Rhapsody™ Sequence Analysis Pipeline. **(B)** UMAP differentiating long COVID patients and controls. **(C)** Bar plots summarizing the number of differentially expressed genes (DEGs) in the various cell types based on a fold change threshold greater than 3 and a *p*-value threshold below 10^−18^.

UMAP clustering of cells revealed distinct expression profiles between patients and controls in classical and intermediate, but not non-classical, monocytes (**Figures 3A, B**). Differential gene expression analysis, based on a fold change threshold greater than 3 and a *p*-value threshold below 10^−18^, identified 40 DEGs in classical, 13 in intermediate, and 6 in non-classical monocytes, while other cell types showed minimal changes (**Figure 3C**).

**Figure 4:**
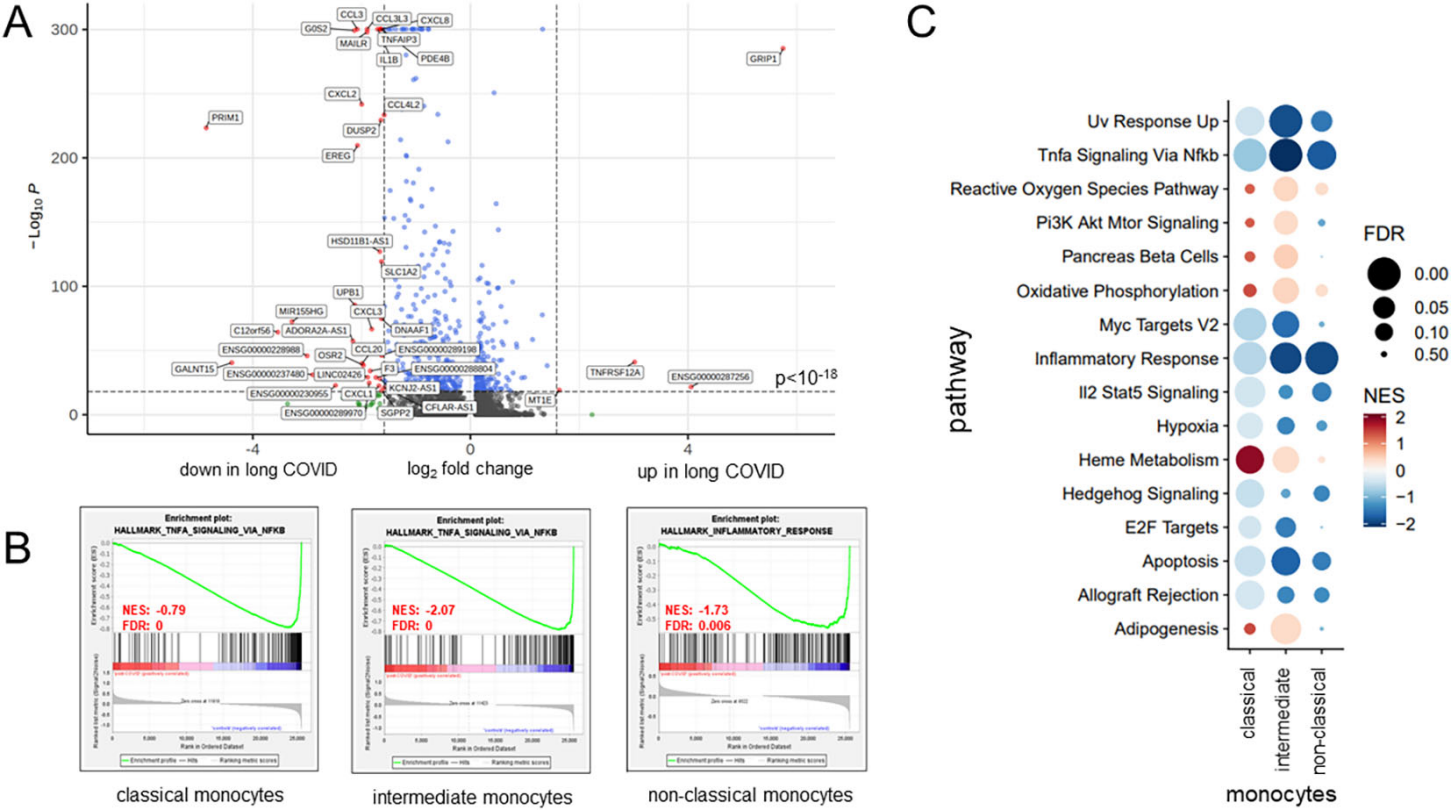
Classical monocytes in long COVID patients showed functional alterations. **(A)** Volcano plot specifying the differentially expressed genes (DEGs) in classical monocytes based on a fold change threshold greater than 3 and a *p*-value threshold of 10^−18^. **(B)** Gene set enrichment analysis results, selected based on a normalized enrichment score (NES) >0 and a false discovery rate (FDR) <0.01, revealing, for patients with long COVID, a significant reduction in the expression of the key gene sets associated with inflammation pathways. **(C)** Bubble plot summarizing, for the various monocyte subsets, the results of further gene set enrichment analyses and showcasing additional pathways that were consistently downregulated in patients. *Color coding* and the *size of the bubbles* indicate FDR and NES, respectively.

The volcano plot in **Figure 4A** details these findings for the classical monocytes. In total, there were 37 downregulated genes in patients with long COVID, among them *IL1B*, several chemokines (CCL3, CCL4, CXCL1, CXCL2, CXCL3, and CXCL8), and the TNFα-induced protein 3 (*TNFAIP3*). Further downregulated genes included *DUSP2*, encoding a phosphatase that downregulates members of the mitogen-activated protein (MAP) kinase superfamily (53), and *MIR155HG*, a microRNA involved in the regulation of MHCII antigen presentation (54) (**Figure 4A**). In order to eliminate the possibility that the observed differences between patients and controls were merely a consequence of the varying intervals between the blood sampling and the most recent SARS-CoV-2 infection, we employed the anti-nucleocapsid titers as a surrogate and correlated them with the respective raw read counts for each of the 40 DEGs. **Supplementary Table S2** presents the correlation coefficients, with range between 0.0133 and 0.185 or −0.0003 and −0.217, thus ruling out any simple linkage. To further evaluate the use of convalescents as a control group, we compared our data to those of publicly available datasets generated via scRNA-seq of healthy controls from two different studies (55, 56). These external control groups were free of immune disorders and, when compared with our long COVID group, confirmed a consistent downregulation of the majority of the DEGs (**Supplementary Figure S2**).

**Figure 5:**
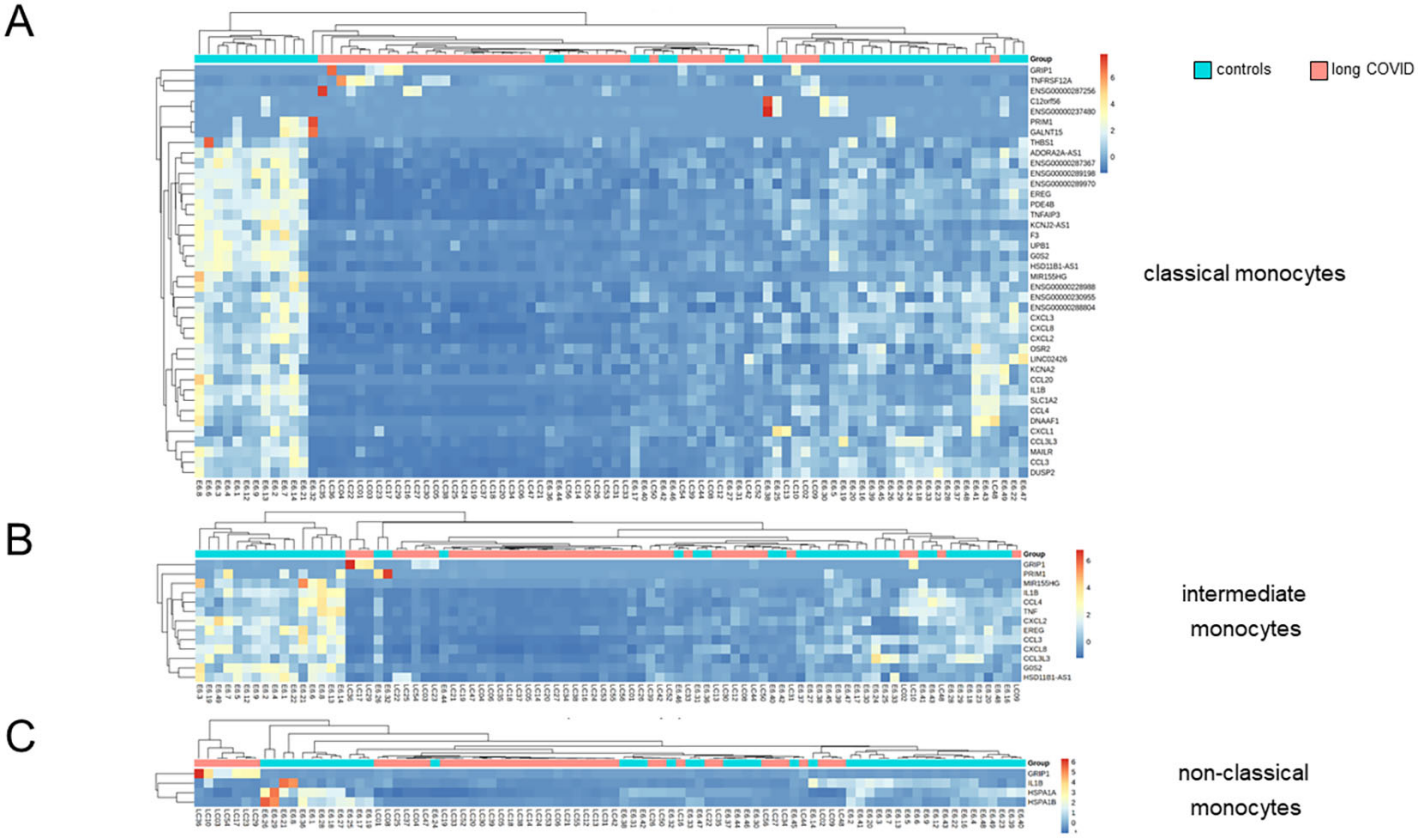
Differentially expressed genes in classical monocytes revealed significant differences between long COVID patients and controls. **(A)** Heatmap showing the results of the Euclidean distance metrics of the single-cell RNA sequencing (scRNA-seq) data from classical monocytes, segregating long COVID patients and controls into three major clusters. **(B, C)** Respective heatmaps for intermediate and non-classical monocytes. Box plots summarizing the median counts per million (CPM)/gene and comparing patients and controls are depicted in **Supplementary Figure S3**.

As monocytes displayed the most DEGs, GSEA was performed for this subset using NES > 0 and FDR < 0.01. Patients with long COVID showed a significantly reduced expression of the inflammation-related pathways, including TNFα signaling via NF-κB, consistently through all subsets of monocytes (**Figure 4B**). Additional pathways, such as inflammatory response and apoptosis, were also downregulated, while heme metabolism and hallmark genes of oxidative phosphorylation (although failing to reach significance) demonstrated a higher NES in patients compared to the controls (**Figure 4C**). Of note is that the downregulated genes in the monocytes of patients formed a homogeneous pattern, clearly separating patients and controls, and most prominently in classical and intermediate monocytes, although to a lower extent also visible in non-classical monocytes (**Figures 5A–C**).

The three most significantly upregulated genes in the classical monocytes of patients with long COVID were *ENSG00000287256*, *GRIP1*, and *TNFRSF12A*, the latter a weak inducer of apoptosis linked to fibrosis-related pathways (57) (**Figure 4A**). However, application of Euclidean distance metrics revealed for the former two an upregulation in only very few patients, while the majority remained inconspicuous (**Figure 5A**). **Supplementary Figure S3** summarizes the differences in the expression patterns between patients and controls based on median counts per million (CPM) per DEG. Consistent with the GSEA results, intermediate monocytes showed significant expression of the genes related to inflammation, such as *IL-1b*, *CXCL2*, and *CXCL8*, with uniformly lower results in patients.

**Figure 6:**
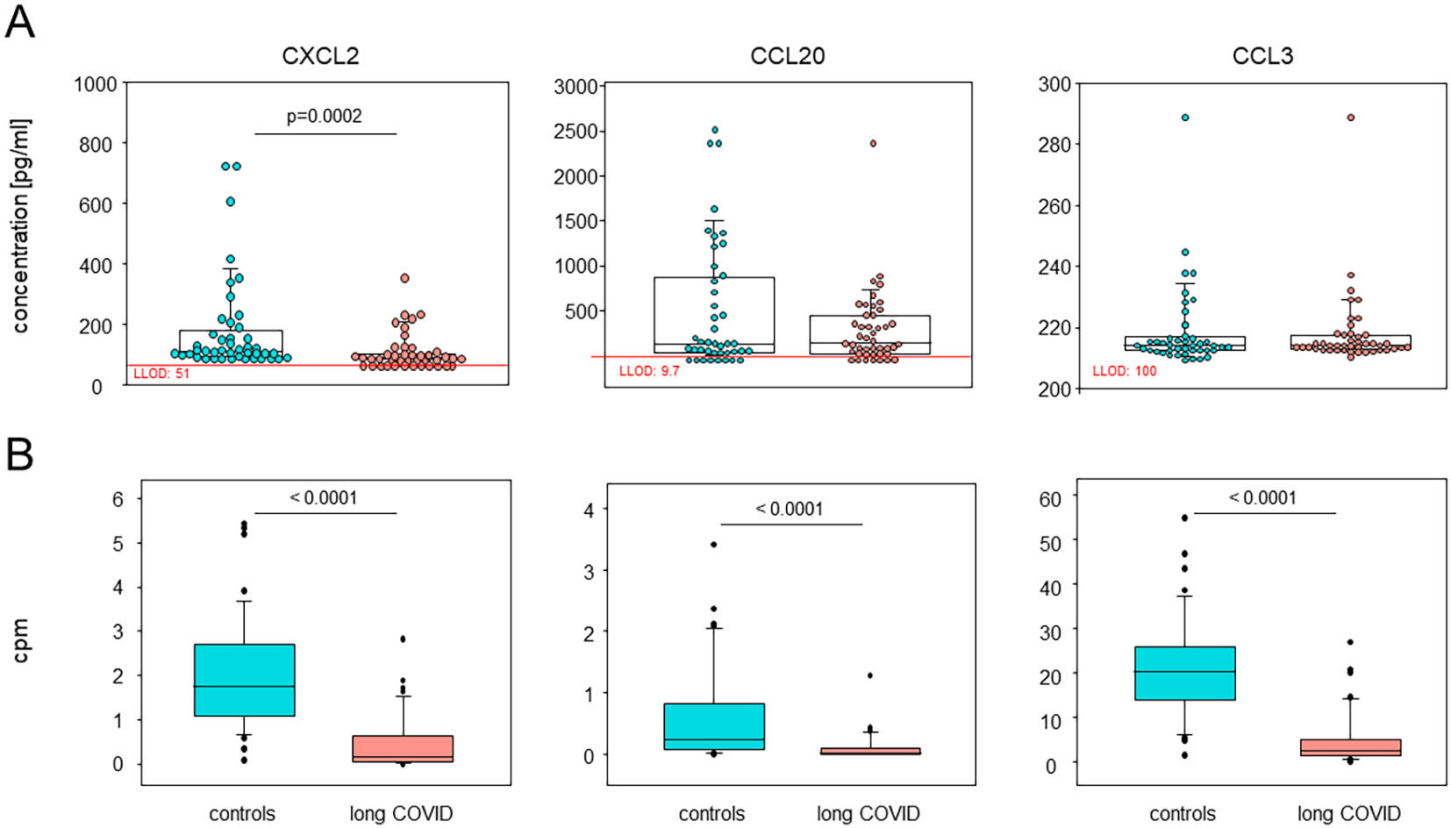
Plasma CXCL2 and CCL20 confirmed the single-cell RNA sequencing (scRNA-seq) data. **(A)** Box plots overlaid with dots representing individual patients and controls summarizing the plasma concentrations of CXCL2, CCL20, and CCL3, analyzed using Luminex assays (*n* = 44 each). *Boxes* represent 25, 50, and 75 percentiles, and *lower* and *upper whiskers* indicate 10 and 90 percentiles, respectively. *P*-values are the results of two-sided Mann–Whitney *U* tests. *LLOD*, lower limit of detection. **(B)** Box plots showing the corresponding differentially expressed genes in all monocytes via the median counts per million (CPM) of patients and controls. *Boxes* represent 25, 50, and 75 percentiles, and *lower* and *upper whiskers* indicate 10 and 90 percentiles, respectively. Outliers are shown. *P*-values are the results of two-sided Mann–Whitney *U* tests.

The plasma concentrations of CXCL2 confirmed the RNA sequencing (RNAseq) data, while other chemokines, such as CCL20, showed a trend that did not quite reach statistical significance (**Figure 6**). The plasma concentrations of IL-6, TNFα, IFNγ, IL-1β, CXCL1, CXCL8, and CCL4 remained below their respective detection thresholds (**Supplementary Table S3**).

As there was a minor quantitative difference observed for the NK cell populations, differential expression of the genes was also analyzed. However, only two genes allowed for the clustering of patients and controls. These genes were identified as TNFα-induced protein 8-like protein 2 (*TNFAIP8L2*) and *CISH*, both of which have been implicated in the regulation of immune processes. Both were downregulated in patients with long COVID (**Supplementary Figure S4**).

In summary, we here showed that the differences in the immune landscape between patients with long COVID and controls were i) restricted to classical and intermediate monocytes; ii) reflected in the DEGs that are predominantly downregulated; and iii) confirmed as reduced protein concentrations in the plasma for CXCL2 and CCL20.

### Differentially expressed genes in the classical monocytes of long COVID patients hint at less inflammasome activity and an immature phenotype

**Figure 7:**
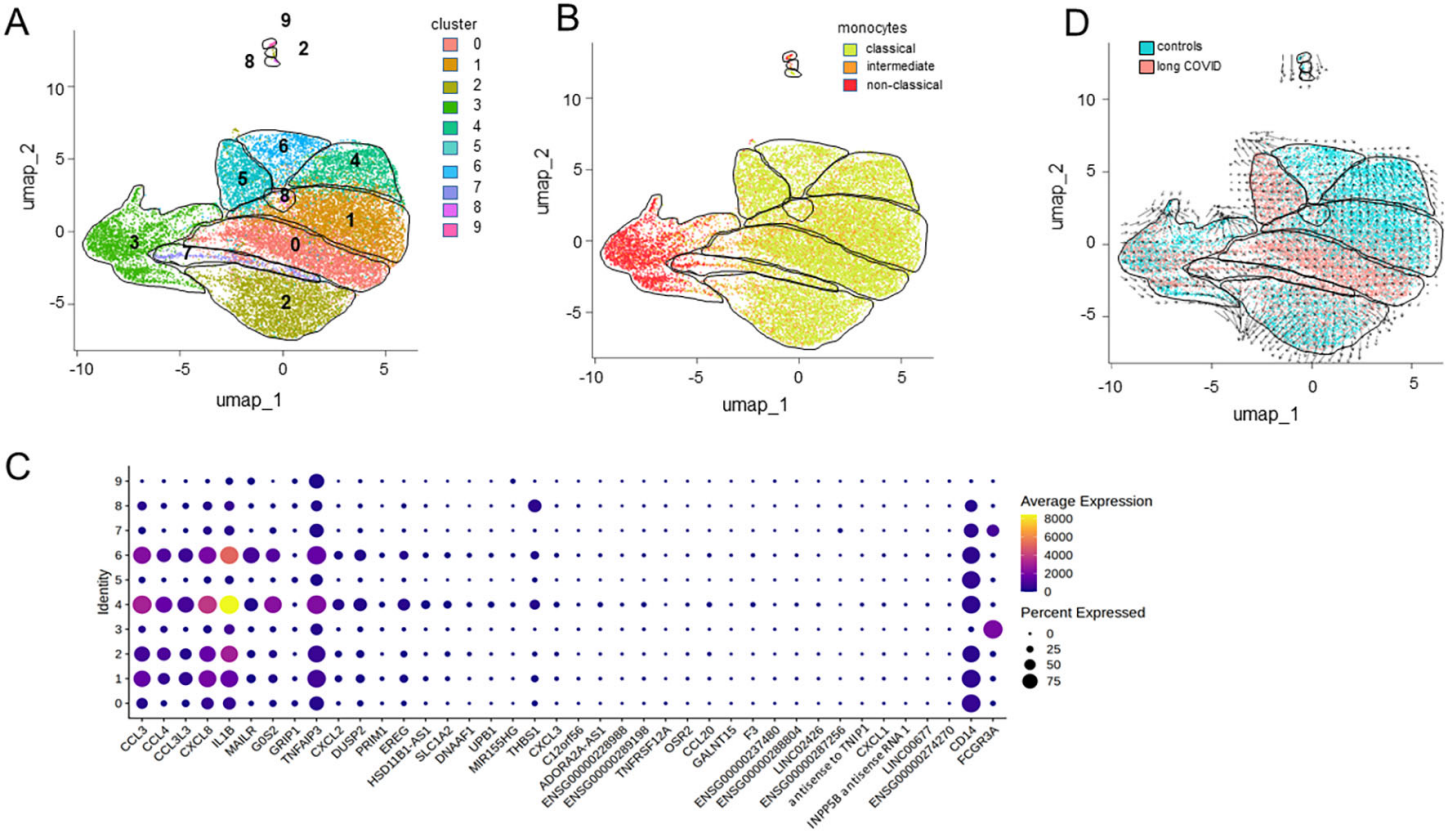
Differentially expressed genes from distinct monocyte subclusters. **(A)** Uniform manifold approximation and projection (UMAP) of the single-cell RNA sequencing (scRNA-seq) data from classical monocytes identifying 10 distinct clusters. **(B)** UMAP differentiating classical from intermediate and non-classical monocytes. **(C)** Bubble heatmap visualizing the gene expression for each cluster, where the *color* and the *dot size* indicate the average scaled expression levels and the percentages of cells expressing the respective gene, respectively. **(D)** RNA velocity trajectories indicating the differentiation of distinct populations of monocytes against the background of patient and control cells.

Subsequent subclustering analysis yielded 10 distinguishable monocyte populations (**Figure 7A**). Subcluster 3 was predominantly associated with non-classical monocytes, as well as a portion of subcluster 7. All the other subclusters were associated with classical and intermediate monocytes (**Figure 7B**). The bubble heatmap in **Figure 7C** provides a detailed visualization of all 40 DEGs, highlighting the subclusters in which each gene was expressed and the proportion of cells within those subclusters expressing the respective genes. **Figure 7D** attributes subclusters 0, 5, and 7 to monocytes from the patients and subclusters 4 and 6 to the controls.

Moreover, the RNA velocity trajectories suggested an origin of differentiation and thereby majority of the immature monocytes in subcluster 0 and neighboring areas that comprise classical and intermediate monocytes from patients in subclusters 1 and 7 (**Figure 7D**). From this origin, the trajectories extended in two directions, either via subcluster 1 toward subcluster 4 or toward subcluster 2. However, interestingly, the trajectories pointed from subcluster 6 toward subcluster 5, suggesting another origin of differentiation. Similarly, the trajectories from subcluster 3, consisting of non-classical monocytes, pointed to various directions, except toward the patients’ monocytes. However, subclusters 3, 5, 7, 8, and 9 harbored the least DEGs (see **Figure 7C**).

**Figure 8:**
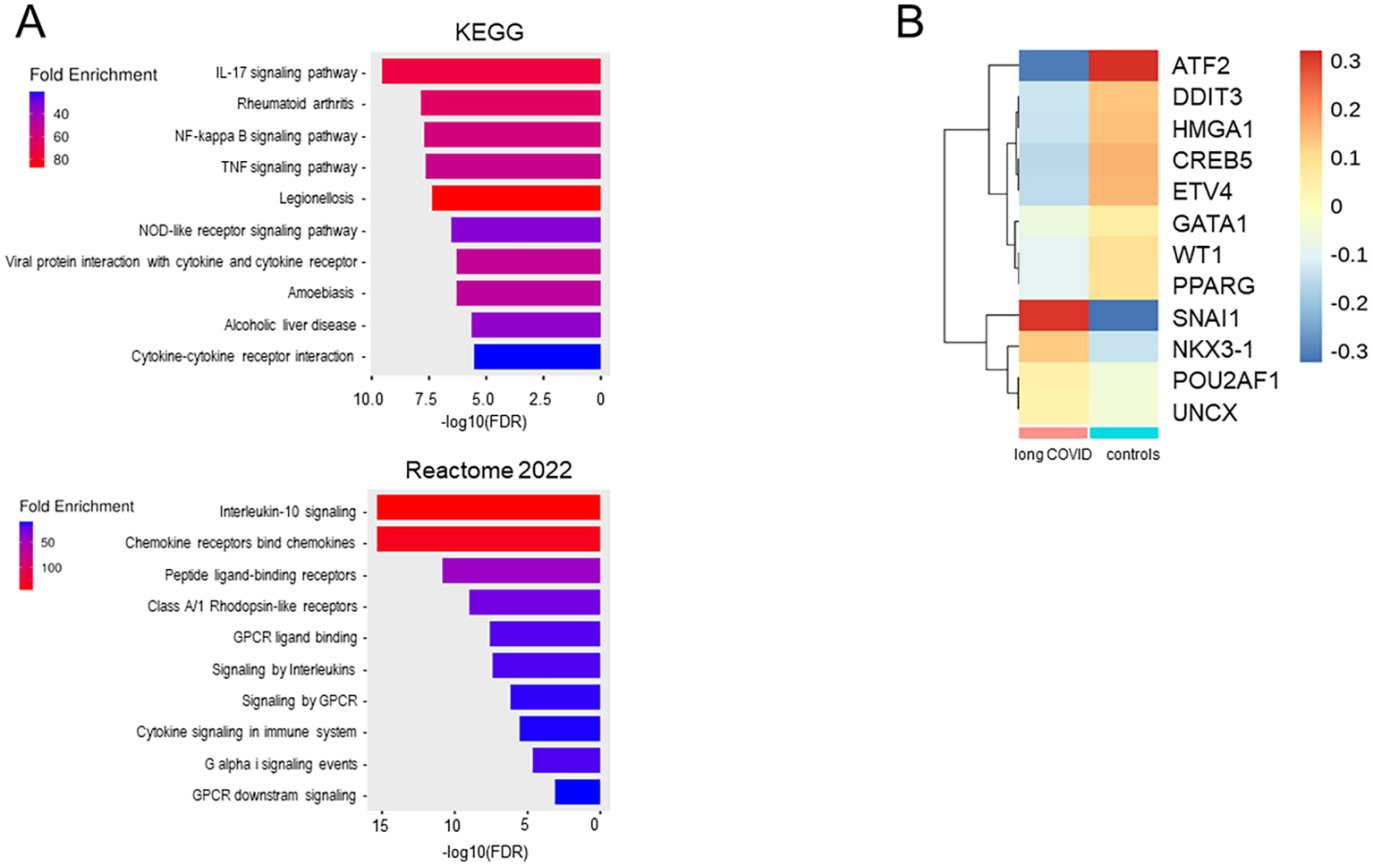
Monocytes from long COVID patients revealed changes in the immunological pathways, transcription factor activities, and differentiation stages. **(A)** Bar plots of the downregulated pathways in long COVID patients created with the ShinyGO software by comparing the downregulated genes (*n* = 37) in all monocytes of the patients and ranked using the false discovery rate (FDR). **(B)** Heatmap showing the transcription factors (TFs), as inferred via DoRothEA, regulating the genes expressed in the monocytes of patients and controls. Plots were generated from the expression data of all monocytes for all genes.

Gene Ontology (GO) enrichment analysis of the 37 downregulated genes in all monocytes from patients, ranked based on the FDR, highlighted functional alterations in long COVID. Cross-referencing with the KEGG and Reactome databases revealed that the infection-related pathways (IL-17, IL-10, and TNFα signaling) were significantly enriched among the downregulated genes, indicating the reduced activity of these pathways in the classical and intermediate monocytes of patients. Similarly, pathways linked to pro-inflammatory conditions such as rheumatoid arthritis and legionellosis were found to be suppressed in patients (**Figure 8A**). To assess transcriptional regulation in patients with long COVID, the TF activities were imputed from the expression data of all the genes expressed in all monocytes using DoRothEA. The heatmap in **Figure 8B** shows that the monocytes from patients appeared to be primarily regulated by *SNAI1*, which is known to suppress inflammasome activity (58). Conversely, *ATF2*, a TF associated with monocyte differentiation (59), displayed a markedly reduced activity in patients. A more extensive spectrum of TFs exhibited weaker differences, encompassing *WT1*, *ATF2*, *ETV4*, *DDIT3*, and *HMGA1*.

In summary, the transcriptional profile of classical monocytes implied not only a lower expression of pro-inflammatory cytokines and various chemokines but also an involvement of TFs that suppress inflammasome activity in patients. A conspicuous deficiency of transcriptional regulation promoting monocyte differentiation was observed, indicating an immature phenotype in the classical monocytes of patients. The RNA velocity data corroborated these findings, as the patient-derived subcluster 0 not only contained the largest number of DEGs but also the least differentiated monocytes.

### Distinctive gene expression in long COVID patients did not correlate with the clinical parameters

In order to explore potential diagnostic indicators for long COVID, we set out to investigate the correlations between routine pathology parameters, quality of life, and the DEGs in classical monocytes. We here concentrated on classical monocytes as they not only expressed the most DEGs but also constituted by far the largest fraction of peripheral monocytes. As demonstrated in **Supplementary Figure S5**, there was no linkage, and neither was there any correlation between the main clinical symptoms and the DEGs.

## Discussion

Our scRNA-seq analysis complemented by plasma profiling provided a comprehensive and novel immune landscape of long COVID syndrome. We revealed qualitative rather than quantitative alterations, confined to classical monocytes, confirming the importance of innate immunity in long COVID (24, 60). Notably, we observed the reduced gene expression of the inflammatory mediators in patients, including *IL1B* and various chemokines, contrasting prior studies that reported a transiently pro-inflammatory monocyte state (25, 28). TF imputation via DoRothEA pointed to an enhanced activity of *SNAI1*, implicating inflammasome suppression, and reduced *ATF2* activity, indicating impaired monocyte-to-macrophage differentiation (58, 59). Together with the RNA velocity analysis, these findings support a developmental impairment in the immature classical monocytes of patients with long COVID. While the proportions of the monocyte subset remained unchanged, we speculate that some classical monocytes still differentiated into intermediate and non-classical monocytes, explaining the decreasing number of DEGs along the maturation trajectory. Functionally, immature classical monocytes in the peripheral blood may lead to a deficit of mature classical macrophages in surrounding tissues, potentially reducing the pathogen defense capability (61). Indeed, anecdotal reports from patients indicated increased susceptibility to infections after having acquired long COVID. Future work should therefore not only investigate the tissue macrophages of patients with long COVID but also monitor in detail seasonal infections.

The question whether the reduced inflammatory potential and the immature phenotype of classical monocytes in patients are a cause or a consequence of long COVID arises. In case of a predisposition, the restricted inflammatory response from macrophages could impair viral control during infection. Indeed, more severe courses of COVID-19 predispose for long COVID (7, 9), and impaired viral control may allow viral persistence, facilitating long COVID (11–13, 62). Although patients did not report immune defects pre-illness, a mild monocytic impairment could explain the observation that, in the majority of cases with long COVID, a single infection with SARS-CoV-2 sufficed triggering long COVID (8). An impaired cytokine production during infection would also delay immune cell recruitment to the lung and thus prevent efficient elimination of the virus, causing damage to the lung and facilitating post-exertional malaise, fatigue, persistent cough, shortness of breath, and chest pain in the long run (63).

Impaired monocyte maturation as a consequence of SARS-CoV-2 infection, on the other hand, is quite intriguing as it implies an innate memory. While the concept of adaptive immune memory is well established, evidence for comparable mechanisms in innate immunity has only been recently recognized. These mechanisms are termed trained immunity or immune tolerance, depending on whether the immune response to a repeated encounter with a pathogen is upregulated or muted (64–68). Indeed, viral infections can induce long-term immune alterations and organ-specific complications (69). Given that monocyte gene expression is still altered more than a year post-infection and that classical monocytes circulate through the periphery for only 1 day before being recruited to the various tissues, an alteration in progenitor cells is strongly indicated (70). Epigenetic reprogramming of the monocytes and hematopoietic progenitors has been described in severe COVID-19, resulting in the hyperactivation of monocytes during infection (71). In long COVID, our data instead suggest a state of immune tolerance. In support of this, another research group demonstrated that stimulation with the SARS-CoV-2 envelope protein induced tolerance in human monocytes, reducing the responses against secondary stimuli (72). A decline in innate and adaptive immune cells has been recently shown in individuals infected with SARS-CoV-2 at 10 months after COVID-19 (69). It remains unclear why some recover while others maintain innate memory and develop sequelae. However, a recent study on the post-acute sequelae of Ebola virus disease has also described a dysregulation of monocytes and thus may confirm shared biological pathways among post-acute infection syndromes (73).

A state of immune exhaustion induced by prolonged viral challenge has been proposed as a mechanism in long COVID. Monocytes from infected individuals have been observed to exhibit a decrease in cytokine secretion, a finding similarly observed in our patient cohort (74). This aligns with previous long COVID studies reporting cytokine deficiencies and suggesting immune exhaustion as a pivotal factor of the disease (63). Exhausted monocytes with an impaired differentiation capacity have also been implicated in the pathogenesis of sepsis (75, 76). Our GSEA only partially supported this hypothesis. We observed a trend toward elevated *OPXHOS* in patients, consistent with exhaustion-associated metabolic reprogramming (77). In contrast, the apoptotic pathways were downregulated, which does not fully align with classical exhaustion patterns (78). Moreover, patients with long COVID showed no elevated expression of PD-1, no reduction in CD86 or MHCII, and no depletion of innate immune cells, indicating that a direct translation of the exhaustion mechanisms seen in other diseases may be limited.

There are additional aspects of our results that are worth discussing. *TNFRSF12A*, a mediator of lung fibrosis in severe COVID-19 (57), was robustly upregulated, suggesting a possible therapeutic target. Secondly, the NK cell alterations regarding relative proportions and expression profiles were minor, but were consistent with previous research that reported a reduced cytotoxic capacity in NK cells during severe SARS-CoV-2 infection, highlighting their potential relevance in the pathogenesis of long COVID (79). Thirdly, we did not find any robust correlation between the gene expression profiles of patients and the clinical assessments, which may indicate that the distinct immunological changes in patients with long COVID contribute to rather diverse phenotypic disease characteristics.

Finally, several limitations have to be acknowledged. Firstly, due to the lack of pre-infection samples, we cannot discriminate whether our results are a cause or a consequence of long COVID. While prospective studies would be ideal, they are becoming increasingly unfeasible as the majority has now been infected with SARS-CoV2. Secondly, convalescents served as controls and were matched for age, sex, and immunization histories. However, as healthcare workers, they had experienced either more frequent or more recent SARS-CoV-2 infections, as indicated by the higher anti-nucleocapsid titers. However, the scRNA-seq results did not correlate with the anti-nucleocapsid titers, supporting our interpretation of a downregulation on the patients’ side rather than an upregulation in the controls. Our findings were further validated by an external healthy control cohort. Thirdly, a long COVID diagnosis remains challenging due to the lack of definitive biomarkers. Even with standardized diagnostic procedures in place at our medical care center, some uncertainties persist. Fourthly, the variants of concern were inferred from the infection time points; however, as the majority of the primary infections in both cohorts occurred predominantly in 2022, Omicron was likely dominant (52). Finally, our focus on PBMCs did not allow any conclusion on transcriptional changes in neutrophils, which may have complemented the picture.

In conclusion, we here present a downregulation of the inflammatory pathways in the classical monocytes of patients with long COVID. We discuss our results as a consequence of a genetic predisposition on the patients’ side, immune exhaustion due to persistent infection, or even epigenetic reprogramming due to SARS-CoV-2. Further *in vitro* research is required to discriminate between the latter two and to explore therapeutic interventions stimulating classical monocytes.

## Data Availability

The datasets presented in this study can be found in online repositories. The names of the repository/repositories and accession number(s) can be found below: https://www.ncbi.nlm.nih.gov/geo/, GSE286325.

## Supplementary material

Supplementary Figure 1 Quality control steps for the assessment of scRNA-seq data. **(A)** Violin plots depict for each experimental run the numbers of different genes detected per cell. Horizontal lines indicate inclusion parameters of cells with <200 or >4000 unique RNA products. **(B)** Violin plots depict for each experimental run the total numbers of reads per cell. **(C)** Violin plots depict for each experimental run the percentages of mitochondrial genes detected per cell. The horizontal line indicates the exclusion of cells with >25% of mitochondrial genes. **(D)** The UMAP represents for each experimental run spike control cells (isolated CD3+ T cells); color code is given. (E+F) Bar charts of cell mixing score and smooth cell mixing score were generated using the CellMixS 1.7.1 package for R and show sufficiently uniform distributions.

Supplementary Figure 2 28 DEGs were confirmed with external controls. Publicly available data sets of controls were compared to the long COVID patients and confirmed 28 down regulated genes in the patients. Box plots summarize normalized and z-scaled expression levels.

Supplementary Figure 3 DEGs mainly consist of down-regulated genes in long COVID patients. The Box-plots compare for each differentially expressed gene in classical **(A)**, intermediate **(B)** and non-classical monocytes the CPM of patients and controls. Boxes represent 25, 50 and 75 percentiles, lower and upper whiskers indicate 10 and 90 percentiles, respectively, outliers are shown. P-values result from two-sided Mann-Whitney U-tests, **p<0.01; ***p<0.001.

Supplementary Figure 4 NK cells also exhibited a down-regulation of differentially expressed genes. **(A)** Volcano plot specifies DEGs in NK cells based on a fold change threshold greater than three and a p-value threshold below 10^-18^. **(B)** The heatmap shows the results of Euclidean-distance metrics of scRNA-seq data from NK cells. Only TNFAIP8L2 and CISH allow for clustering of patients and controls. **(C)** The Box-plots compare for TNFAIP8L2 and CISH the CPM in NK cells of patients and controls. Boxes represent 25, 50 and 75 percentiles, lower and upper whiskers indicate 10 and 90 percentiles, respectively, outliers are shown. P-values result from two-sided Mann-Whitney U-tests.

Supplementary Figure 5 Distinctive gene expression in long COVID patients did not correlate with clinical parameters. Heatmaps show the results of Euclidean-distance metrics of scRNA-seq data from classical monocytes and routine pathology parameters, clinical symptoms and quality of life, respectively **(A)** and patients’ predominant clinical symptoms **(B)**. Color codes for predominant clinical symptoms of each patient are specified.
