# Supplementary figures and images for: Transcriptome analysis of classical blood cells reveals downregulation of pro-inflammatory genes in the classical monocytes of long COVID patients
Source: Front Immunol. 2025 Nov 7;16:1710783. doi: 10.3389/fimmu.2025.1710783 (PMC12634634; doi:10.3389/fimmu.2025.1710783)

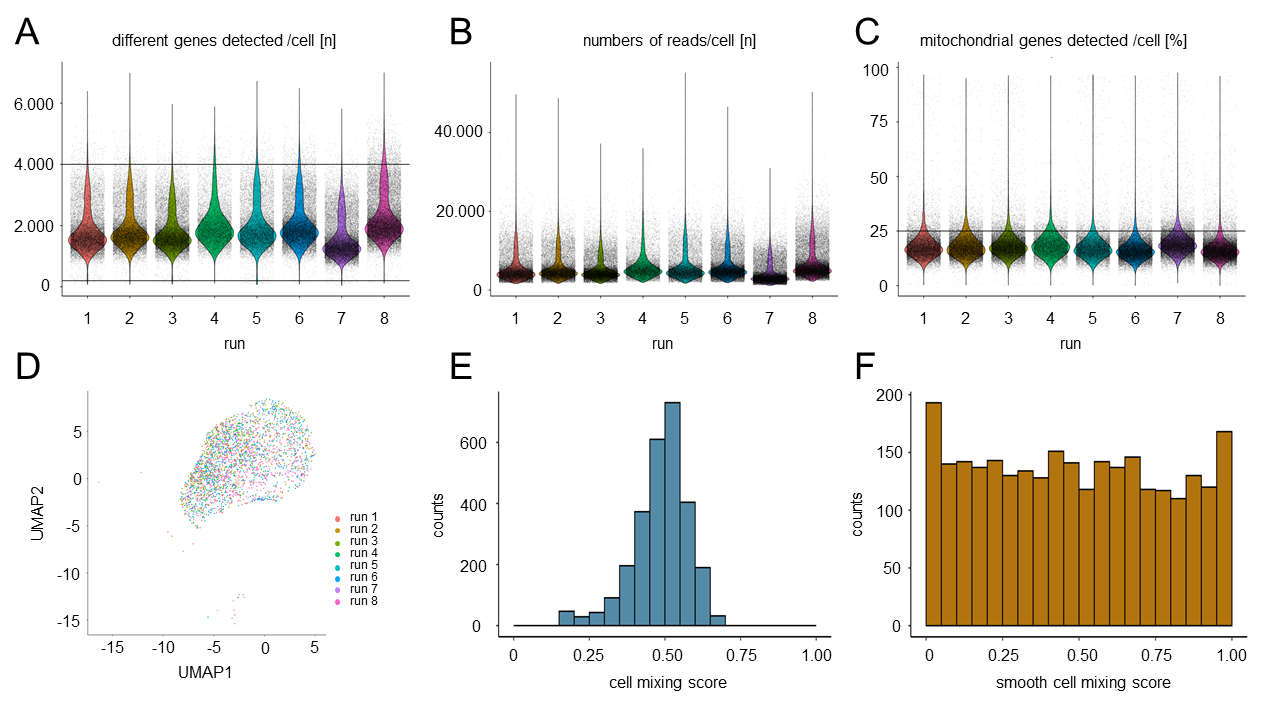

Supplement: Supplementary Figure 1 — Quality control steps for the assessment of scRNA-seq data. (A) Violin plots depict for each experimental run the numbers of different genes detected per cell. Horizontal lines indicate inclusion parameters of cells with <200 or >4000 unique RNA products. (B) Violin plots depict for each experimental run the total numbers of reads per cell. (C) Violin plots depict for each experimental run the percentages of mitochondrial genes detected per cell. The horizontal line indicates the exclusion of cells with >25% of mitochondrial genes. (D) The UMAP represents for each experimental run spike control cells (isolated CD3+ T cells); color code is given. (E+F) Bar charts of cell mixing score and smooth cell mixing score were generated using the CellMixS 1.7.1 package for R and show sufficiently uniform distributions. [file Image1.tif]

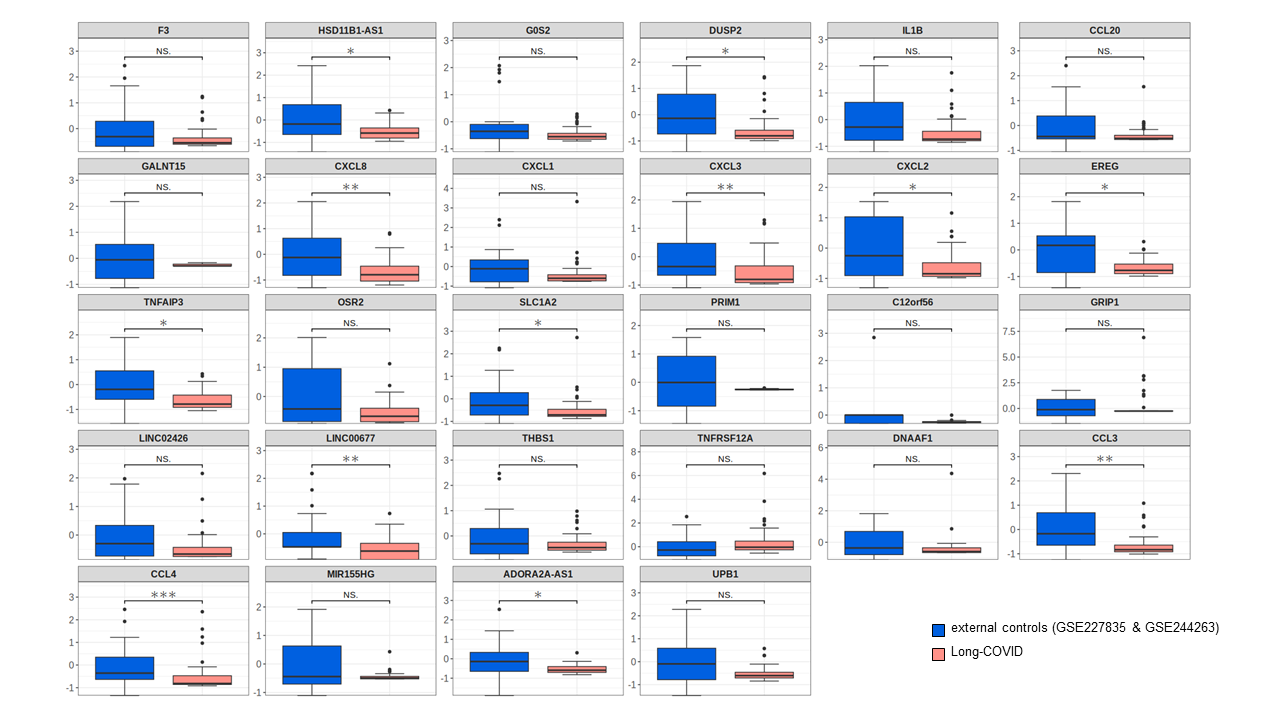

Supplement: Supplementary Figure 2 — 28 DEGs were confirmed with external controls. Publicly available data sets of controls were compared to the long COVID patients and confirmed 28 down regulated genes in the patients. Box plots summarize normalized and z-scaled expression levels. [file Image2.tif]

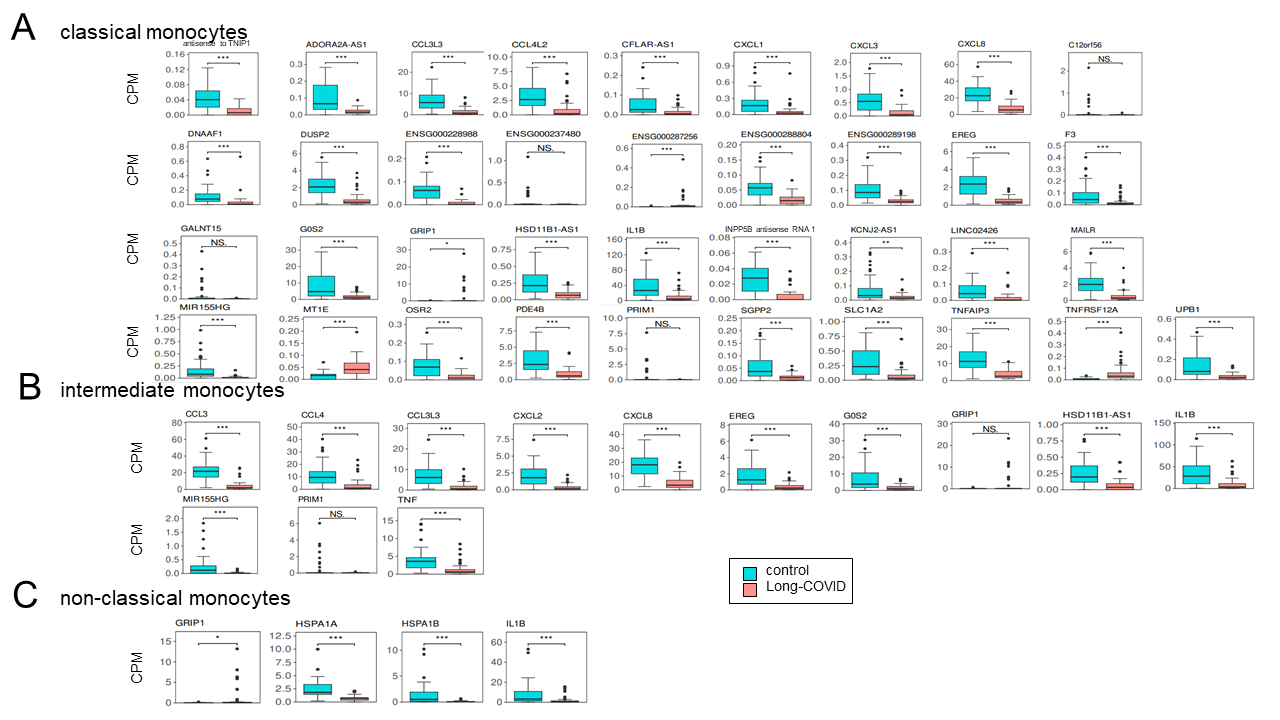

Supplement: Supplementary Figure 3 — DEGs mainly consist of down-regulated genes in long COVID patients. The Box-plots compare for each differentially expressed gene in classical (A), intermediate (B) and non-classical monocytes the CPM of patients and controls. Boxes represent 25, 50 and 75 percentiles, lower and upper whiskers indicate 10 and 90 percentiles, respectively, outliers are shown. P-values result from two-sided Mann-Whitney U-tests, **p<0.01; ***p<0.001. [file Image3.tif]

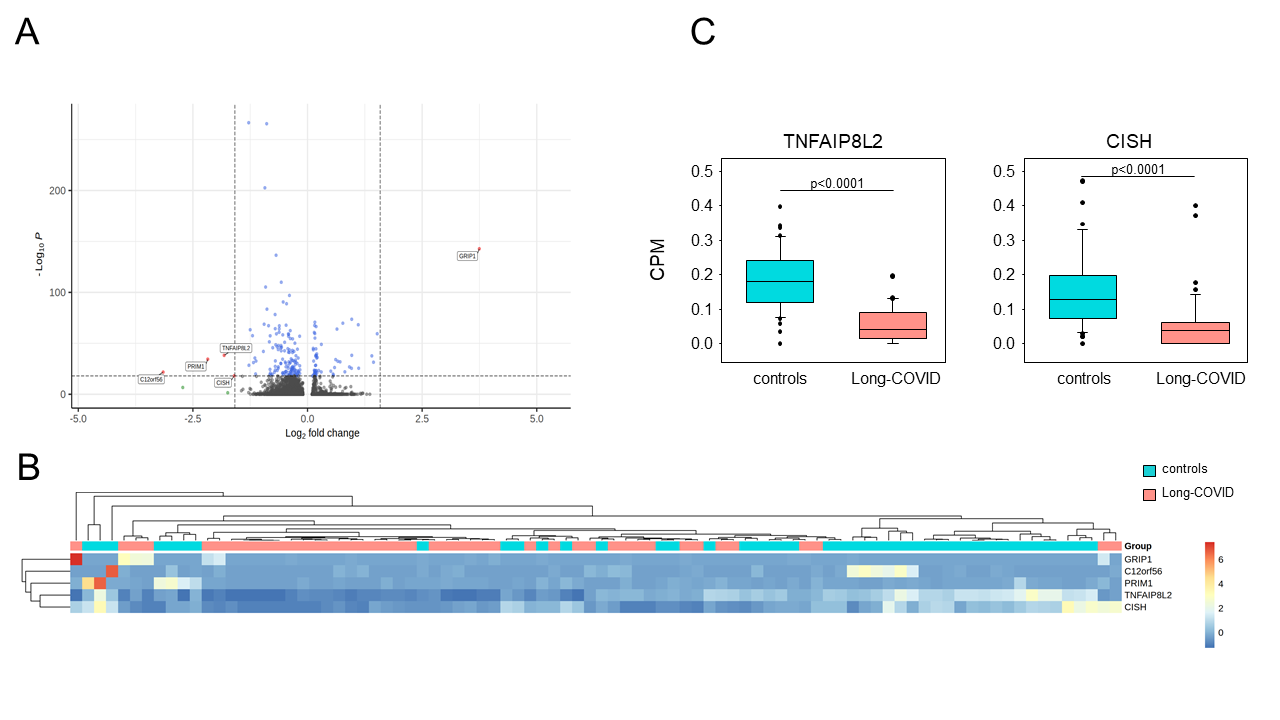

Supplement: Supplementary Figure 4 — NK cells also exhibited a down-regulation of differentially expressed genes. (A) Volcano plot specifies DEGs in NK cells based on a fold change threshold greater than three and a p-value threshold below 10-18. (B) The heatmap shows the results of Euclidean-distance metrics of scRNA-seq data from NK cells. Only TNFAIP8L2 and CISH allow for clustering of patients and controls. (C) The Box-plots compare for TNFAIP8L2 and CISH the CPM in NK cells of patients and controls. Boxes represent 25, 50 and 75 percentiles, lower and upper whiskers indicate 10 and 90 percentiles, respectively, outliers are shown. P-values result from two-sided Mann-Whitney U-tests. [file Image4.tif]

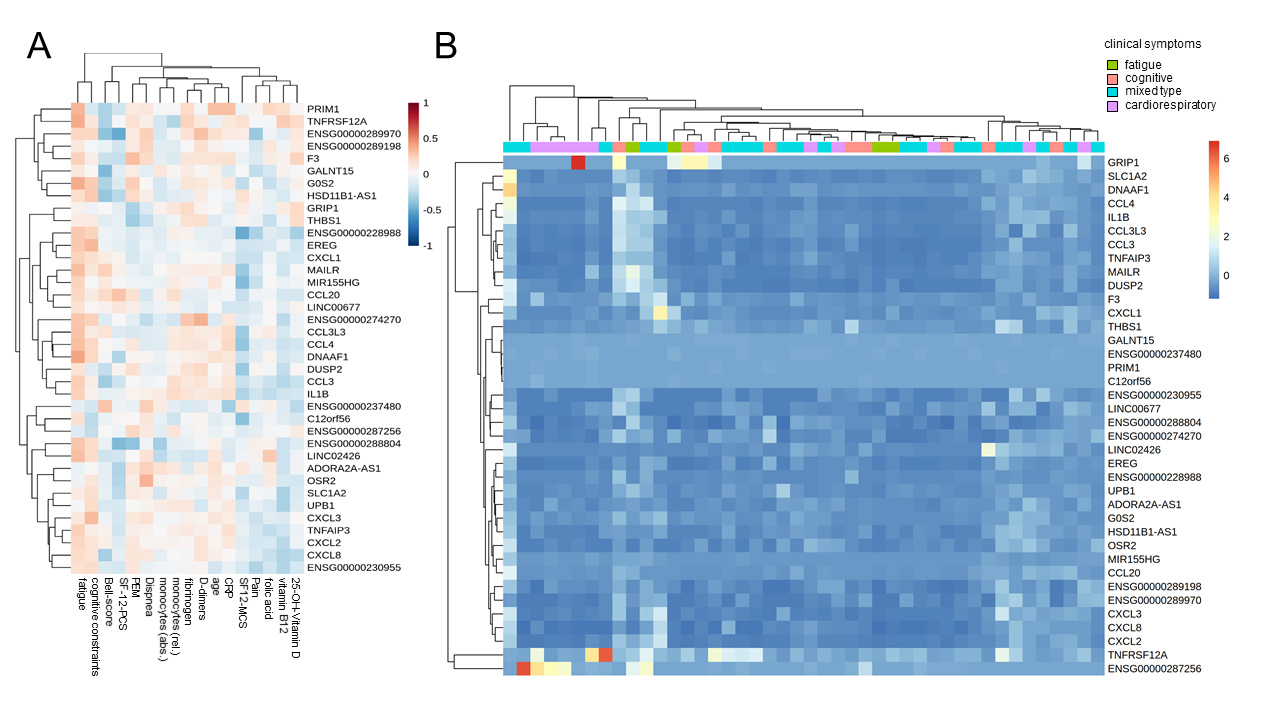

Supplement: Supplementary Figure 5 — Distinctive gene expression in long COVID patients did not correlate with clinical parameters. Heatmaps show the results of Euclidean-distance metrics of scRNA-seq data from classical monocytes and routine pathology parameters, clinical symptoms and quality of life, respectively (A) and patients’ predominant clinical symptoms (B). Color codes for predominant clinical symptoms of each patient are specified. [file Image5.tif]
