# Supplementary material for: Transcriptome analysis of classical blood cells reveals downregulation of pro-inflammatory genes in the classical monocytes of long COVID patients
Source: Front Immunol. 2025 Nov 7;16:1710783. doi: 10.3389/fimmu.2025.1710783 (PMC12634634; doi:10.3389/fimmu.2025.1710783)

**Suppl. Table 1: detailed patient information**

|  |  |  | **symptoms** | | | | | | | | |  |  |  | **Comorbidities (ICD-10)** | | | | | | | | | | | | | |  | **mental/physical health** | | |  |  |  |
| --- | --- | --- | --- | --- | --- | --- | --- | --- | --- | --- | --- | --- | --- | --- | --- | --- | --- | --- | --- | --- | --- | --- | --- | --- | --- | --- | --- | --- | --- | --- | --- | --- | --- | --- | --- |
| patient ID | sex | age (years) | fatigue | cardio_respiratory | cognitive | gastro intestinal | anxiety / depression | pain | loss of sense of smell/taste | dizziness | insomnia | post-exertional malaise | symptom type | severity of COVID-19^29^ | arterial hypertension (I10) | type 2 diabetes mellitus (E11) | osteoarthritis (M15-M19) | thyroiditis (E06) | Graves' disease (E05.0) | obesity (E66) | migraine (G43) | systemic lupus erythematosus (M32) | bronchial asthma (J45) | restless legs syndrome (G25.81) | polyneuropathy (G62.9) | dDepression (F32) | hypothyroidism (E03) | other | treatments following COVID-19 | Bell-Score | mental health (Short-Form 12) | physical health (Short-Form 12) |  |  |  |
| 1 | m | 54 | 2 | 3 | 0 | 0 | 0 | 0 | 2 | 0 | 3 | 0 | cr | mi |  |  |  |  |  |  |  |  |  |  |  |  |  |  | ot | 75 | 26.4 | 63.9 |  |  |  |
| 2 | f | 57 | x | 0 | 0 | 0 | 0 | x | 0 | 0 | 0 |  | f | mi | x |  | x |  |  |  |  |  |  |  |  |  |  | rd | ht/ns |  |  |  |  |  |  |
| 3 | f | 39 | 3 | x | 2 | 0 | 0 | x | 0 | 1 | 3 | x | co | mo | x | x |  | x |  | x | x |  |  |  |  |  |  |  | ns |  | 48.0 | 47.3 |  |  |  |
| 4 | f | 37 | x | x | x | 0 | 0 | x | 0 | x | 0 |  | mt | mi |  |  |  |  |  |  |  | x |  |  |  |  |  |  |  |  |  |  |  |  |  |
| 5 | f | 58 | 3 | 3 | 3 | 0 | 0 | 0 | 1 | 1 | 2 | 0 | mt | mi |  |  | x |  |  |  |  |  | x | x | x |  |  |  | rt | 35 |  |  |  |  |  |
| 6 | f | 59 | 2 | 1 | 3 | 2 | 0 | 0 | 0 | 2 | 3 | 0 | mt | mi | x |  |  |  |  |  |  |  |  |  |  | x |  |  | rt |  | 37.4 | 25.9 |  |  |  |
| 8 | f | 48 | 3 | 1 | 3 | 0 | 2 | 0 | x | 0 | 0 | x | mt | mi | x |  |  |  |  |  |  |  |  |  |  |  |  | cd | d | 25 |  |  |  |  |  |
| 9 | f | 31 | 2 | 2 | 2 | 0 | 2 | 0 | 1 | 0 | 2 | 0 | mt | mi | x |  |  |  |  | x |  |  |  |  |  | x |  | li | rs |  | 22.6 | 30.1 |  |  |  |
| 10 | m | 29 | 2 | 0 | 2 | 0 | 0 | 0 | 3 | 2 | 0 | 0 | co | mi |  |  |  |  |  |  |  |  |  |  |  |  |  |  | ox |  |  |  |  |  |  |
| 12 | f | 53 | 3 | 0 | 3 | 1 | 0 | 2 | 0 | 0 | 3 | 0 | mt | mi |  |  |  |  |  |  |  |  |  |  |  | x |  |  |  |  |  |  |  |  |  |
| 13 | f | 61 | 2 | 2 | 2 | 1 | 1 | 0 | 2 | 2 | 1 | 0 | mt | mi | x |  |  |  |  | x |  |  | x |  |  |  |  | cps |  | 40 | 56. | 27.5 |  |  |  |
| 14 | f | 23 | x | x | x | 0 | 0 | x | 0 | 0 | 0 |  | mt | mo |  |  |  |  |  |  | x |  |  |  |  |  |  |  | rt/c |  |  |  |  |  |  |
| 16 | f | 32 | 0 | x | 0 | 0 | 0 | 0 | 0 | x | 0 |  | cr | mi |  |  |  |  |  |  |  |  |  |  |  |  |  |  | v |  |  |  |  |  |  |
| 17 | f | 55 | x | 0 | x | 0 | 0 | x | 0 | 0 | 0 |  | co | uk |  |  | x |  | x |  |  |  |  |  |  |  |  |  | rt |  |  |  |  |  |  |
| 18 | f | 58 | x | x | x | 0 | 0 | x | 0 | 0 | 0 |  | mt | mo |  |  |  |  |  | x |  |  | x | x |  | x |  | ri | rt |  | 54.6 | 11.7 |  |  |  |
| 19 | f | 30 | 0 | x | 0 | 0 | 0 | 0 | 0 | 0 | 0 |  | cr | mo |  |  |  |  |  |  |  |  |  |  |  |  |  | rs | rt |  |  |  |  |  |  |
| 20 | f | 39 | x | 0 | 0 | 0 | 0 | x | 0 | 0 | 0 |  | f | mi |  |  |  | x |  |  |  |  |  |  |  |  |  |  |  | 40 |  |  |  |  |  |
| 21 | f | 38 | 2 | 0 | 3 | 0 | 0 | 2 | 0 | 0 | 0 | 0 | co | mi |  |  |  |  |  |  |  |  |  |  |  |  |  |  |  | 50 |  |  |  |  |  |
| 22 | f | 54 | 3 | 3 | 0 | 0 | 3 | 2 | 0 | 0 | 3 | x | cr | sev |  |  |  |  |  |  |  |  | x |  |  | x |  | fV | rt/ps | 0 |  |  |  |  |  |
| 23 | f | 51 | 1 | 0 | 0 | 0 | 0 | x | 0 | 0 | 0 | 0 | f | mi | x | x |  |  |  |  |  |  |  |  |  |  |  |  | rt/lt | 60 | 40.5 | 54.1 |  |  |  |
| 24 | m | 55 | 3 | 0 | 3 | 0 | 0 | 0 | 0 | 0 | 0 | 0 | co | mi |  |  |  |  |  |  |  |  |  |  |  |  |  |  | rt | 66 | 33.8 | 58.4 |  |  |  |
| 25 | f | 32 | 0 | x | x | 0 | 0 | x | 0 | 0 | 0 |  | mt | mo |  |  |  |  |  |  |  |  |  |  |  | x |  |  | c |  |  |  |  |  |  |
| 26 | m | 38 | 2 | 0 | 2 | 0 | 0 | 0 | 0 | 0 | 0 | 0 | co | mi |  |  |  |  |  |  |  |  |  |  |  |  |  |  |  |  |  |  |  |  |  |
| 27* | m | 69 | 3 | 3 | 0 | 0 | 2 | 0 | 0 | 0 | 0 | x | cr | cri | x | x |  |  | x |  |  |  |  |  |  |  |  | mcl | rt |  |  |  |  |  |  |
| 29 | m | 61 | 3 | 3 | 0 | 0 | 2 | 2 | 0 | 0 | 2 | 0 | cr | mi | x |  |  |  |  | x |  |  |  |  |  |  |  |  | rt | 35 | 24.5 | 27.7 |  |  |  |
| 30 | m | 50 | 2 | 1 | 3 | 0 | 0 | 0 | 0 | 0 | 0 | 0 | mt | mo |  |  |  |  |  |  |  |  |  |  |  |  |  |  | rt |  | 53.0 | 41.2 |  |  |  |
| 31 | f | 63 | x | x | 0 | 0 | 0 | x | 0 | 0 | 0 |  | cr | mo | x |  | x |  |  |  |  |  |  |  |  |  | x | li |  |  | 57.5 | 23.0 |  |  |  |
| 33 | f | 53 | 3 | x | 3 | 0 | 0 | 2 | 0 | 2 | 0 | 0 | mt | uk |  |  |  |  |  |  | x |  |  |  |  | x |  |  |  |  |  |  |  |  |  |
| 34 | f | 63 | x | x | x | 0 | 0 | x | x | 0 | 0 |  | mt | mi |  |  |  |  |  |  | x |  | x |  |  |  |  |  | rt/ps |  | 48.7 | 32.6 |  |  |  |
| 35 | f | 54 | x | x | x | x | 0 | x | 0 | 0 | 0 |  | mt | mo |  |  |  | x |  |  |  |  |  |  |  |  |  |  | rt/ns |  |  |  |  |  |  |
| 36 | f | 36 | 0 | 3 | 0 | 2 | 2 | 3 | 0 | 0 | 3 | 0 | cr | mi |  |  | x |  |  |  |  |  |  |  |  |  |  |  |  |  |  |  |  |  |  |
| 37 | f | 45 | 3 | 0 | 0 | 1 | 0 | 2 | 0 | 0 | 2 | 0 | f | mo |  |  |  |  |  |  |  |  |  |  |  |  | x |  |  |  |  |  |  |  |  |
| 38 | f | 54 | 2 | 3 | 3 | 0 | 0 | 0 | 2 | 0 | 0 | 0 | mt | mo |  |  |  |  |  |  |  |  |  |  |  |  |  | mcf |  |  | 56.37 | 27.64 |  |  |  |
| 39 | f | 60 | 3 | 3 | 0 | x | 0 | 1 | 0 | 0 | 0 | x | cr | mi | x |  |  |  |  |  |  |  |  |  |  |  | x |  |  | 70 | 33.67 | 65.24 |  |  |  |
| 42 | f | 57 | 3 | 0 | 3 | 2 | 3 | 2 | 0 | 0 | 2 | 0 | co | mi |  |  |  |  |  |  |  |  |  |  |  |  |  | pv |  | 60 | 28.98 | 49.41 |  |  |  |
| 44 | m | 61 | 3 | 3 | 3 | 0 | 3 | 0 | 0 | 0 | 0 | 0 | mt | mo | x | x | x |  |  | x | x |  |  |  |  |  |  |  | rt |  | 31.91 | 34.23 |  |  |  |
| 47 | f | 47 | 3 | 2 | 2 | 0 | 1 | 0 | 3 | 0 | 1 | x | mt | mo |  |  |  |  |  |  |  |  |  |  |  |  | x |  | rt/v | 55 | 48.01 | 47.34 |  |  |  |
| 48 | f | 45 | 3 | 2 | 3 | 0 | 2 | 3 | 1 | 2 | 2 | x | mt | mi |  |  |  |  |  |  |  |  | x |  |  |  |  |  | rt | 30 | 30.17 | 20.45 |  |  |  |
| 50 | f | 51 | x | 0 | x | 0 | 0 | x | 0 | 0 | x |  | co | mi | x |  |  |  |  |  | x |  |  |  |  |  | x | mi |  |  |  |  |  |  |  |
| 52 | f | 55 | 3 | 1 | 3 | 0 | 1 | 2 | 0 | 0 | 2 | 0 | mt | mi |  |  |  |  |  |  |  |  |  |  |  |  |  |  | rt/p |  |  |  |  |  |  |
| 53 | f | 19 | x | 0 | x | 0 | 0 | 0 | 0 | 0 | 2 |  | co | mi |  |  |  |  |  |  |  |  |  |  |  |  |  |  |  |  |  |  |  |  |  |
| 54 | f | 70 | 2 | 3 | 0 | 0 | 0 | 0 | 0 | 1 | 0 | 0 | cr | mo | x |  |  |  |  | x |  |  |  |  |  |  | x |  | rt | 50 | 61.56 | 26.37 |  |  |  |
| 55 | m | 74 | 3 | 3 | 0 | 0 | 0 | 0 | 0 | x | 0 | 0 | cr | mi | x |  |  |  |  |  |  |  | x |  |  |  |  |  | c | 50 | 56.81 | 37.99 |  |  |  |
| 56 | m | 54 | 3 | 3 | 3 | 0 | 2 | 0 | 1 | 0 | 3 | 0 | mt | mi | x |  |  |  |  |  |  |  |  |  |  |  | x | t1d | c/ns/pt/p |  | 27.76 | 28.78 |  |  |  |

*intensive care treatment during COVID-19

Symptom types: co – cognitive; cr – cardio respiratory; f – fatigue; mt – mixed type;

Symptom severity: 0 – absent; 1- mild; 2 – moderate; 3 – severe; x – symptom present, severity not classified; empty cells indicate no data available

Severity of COVID-19: mi- mild; mo – moderate; sev – severe; cri – critical; uk – unknown;

Comorbidities (other): rd - reflux disease(K21); cd - carotid dissection (I72.4); li - lipedema (E88.2); cps - chronic pain syndrome (R52.2); ri - renal insufficiency (N18); rs - Raynaud syndrome (I73.0); attention deficit/hyperactivity disorder (F90); fV - factor V Leiden (D68.5); mcl - mantle cell lymphoma (C83.1); mcf - ME/CFS (G93.3); pv - psoriasis vulgaris (L40.0); mi - myocardial infarction (I21); t1d - type 1 diabetes mellitus (E10);

Treatments following COVID-19: c – cortison; ht – heat treatment; lt – laser therapy; ns – nutritional supplements; ot – osteopathic treatment; ox – hyperbaric oxygen; p – pregabalin; ps – psychotherapy; pt – physiotherapy; rt – respiratory therapy; v – vitamins;


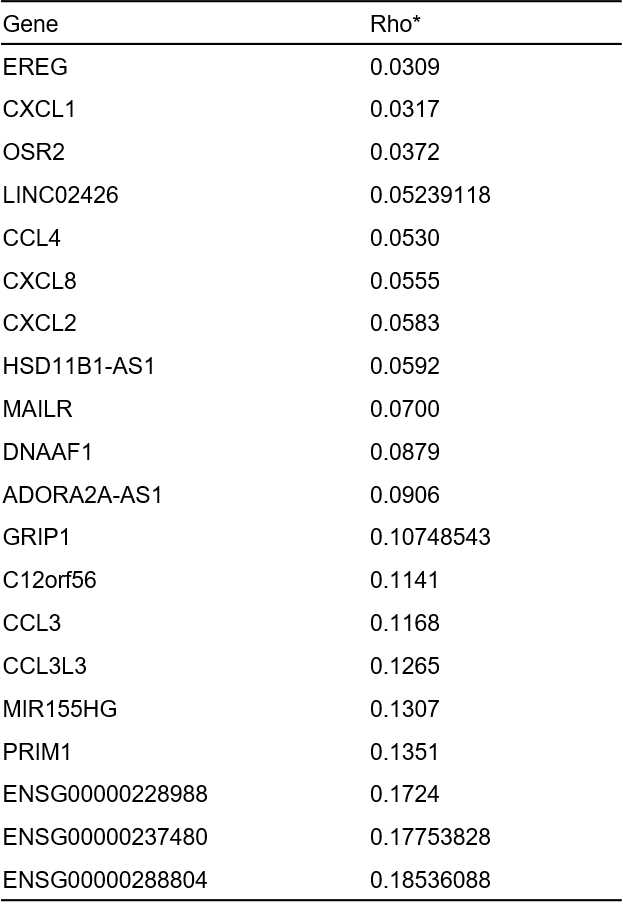

Supplement: Supplementary file 6 [file Table1.docx]
