# Supplementary material for: Transcriptome analysis of classical blood cells reveals downregulation of pro-inflammatory genes in the classical monocytes of long COVID patients
Source: Front Immunol. 2025 Nov 7;16:1710783. doi: 10.3389/fimmu.2025.1710783 (PMC12634634; doi:10.3389/fimmu.2025.1710783)
Supplement: Supplementary file 7 [file Table2.docx]

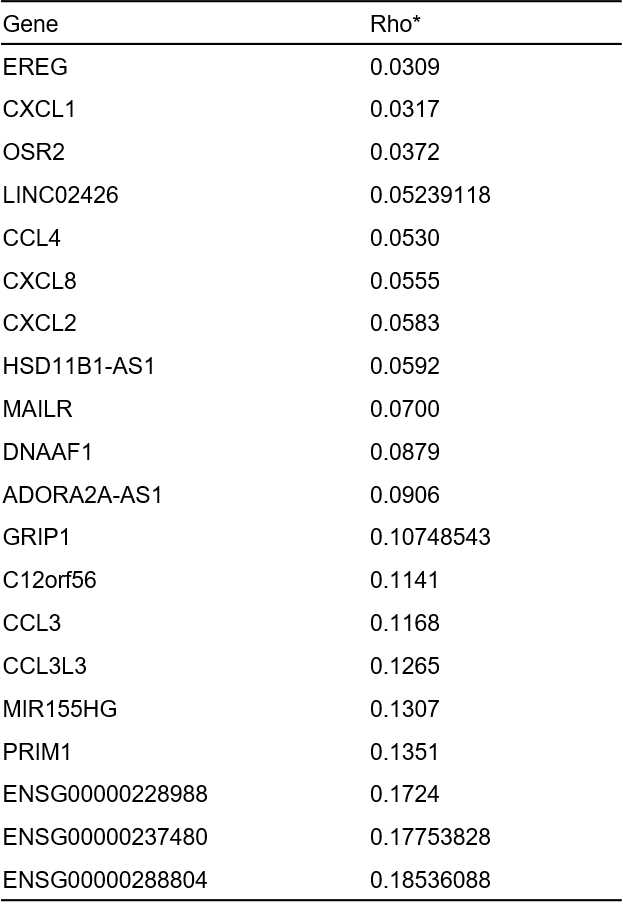


| Gene | Rho* |  | Gene | Rho* |
| --- | --- | --- | --- | --- |
| EREG | 0.0309 |  | EREG | 0.0309 |
| CXCL1 | 0.0317 |  | CXCL1 | 0.0317 |
| OSR2 | 0.0372 |  | OSR2 | 0.0372 |
| LINC02426 | 0.05239118 |  | LINC02426 | 0.05239118 |
| CCL4 | 0.0530 |  | CCL4 | 0.0530 |
| CXCL8 | 0.0555 |  | CXCL8 | 0.0555 |
| CXCL2 | 0.0583 |  | CXCL2 | 0.0583 |
| HSD11B1-AS1 | 0.0592 |  | HSD11B1-AS1 | 0.0592 |
| MAILR | 0.0700 |  | MAILR | 0.0700 |
| DNAAF1 | 0.0879 |  | DNAAF1 | 0.0879 |
| ADORA2A-AS1 | 0.0906 |  | ADORA2A-AS1 | 0.0906 |
| GRIP1 | 0.10748543 |  | GRIP1 | 0.10748543 |
| C12orf56 | 0.1141 |  | C12orf56 | 0.1141 |
| CCL3 | 0.1168 |  | CCL3 | 0.1168 |
| CCL3L3 | 0.1265 |  | CCL3L3 | 0.1265 |
| MIR155HG | 0.1307 |  | MIR155HG | 0.1307 |
| PRIM1 | 0.1351 |  | PRIM1 | 0.1351 |
| ENSG00000228988 | 0.1724 |  | ENSG00000228988 | 0.1724 |
| ENSG00000237480 | 0.17753828 |  | ENSG00000237480 | 0.17753828 |
| ENSG00000288804 | 0.18536088 |  | ENSG00000288804 | 0.18536088 |

**Suppl. Table 2. counts per million (CPM) of differentially expressed genes (DEG) were independent of the respective N-antibodies titers**
