# Supplementary material for: Transcriptome analysis of classical blood cells reveals downregulation of pro-inflammatory genes in the classical monocytes of long COVID patients
Source: Front Immunol. 2025 Nov 7;16:1710783. doi: 10.3389/fimmu.2025.1710783 (PMC12634634; doi:10.3389/fimmu.2025.1710783)
Supplement: Supplementary file 8 [file Table3.docx]

**Suppl. Table 3. plasma cytokine and chemokine concentrations**

| cytokine / chemokine | controls | post-COVID | p-value* |
| --- | --- | --- | --- |
|  | median (min / max) [pg/ml] | |  |
| TNFa | 6.91 (6.91 / 48.09) | 6.91 (6.91 / 48.09) | 0.3258 |
| IL-6 | 3.70 (3.70 / 23.92) | 3.70 (3.70 / 113.60) | 0.6066 |
| IL-8 | 3.95 (3.95 / 17.51) | 3.95 (3.95 / 408.44 | 0.1151 |
| CXCL2 | 112.39 (84.04 / 720.16) | 91.66 (81.50 / 351.17) | **0.0002** |
| IL-1b | 17.33 (17.33 / 33.19) | 17.33 (17.33 / 33.19) | 0.9871 |
| IFNg | 2.39 (2.39 / 8.45) | 2.39 (2.39 / 15.89) | 0.5592 |
| CCL20 | 136.17 (9.71 / 2509.85) | 147.07 (9.71 / 2360.00) | 0.6846 |
| CCL3 | 214.10 ( 209.12 / 288.62) | 214.10 (210.23 / 288.62) | 0.6820 |
| CCL4 | 130.25 (130.25 / 170.64) | 130.25 (130.25 / 170.64) | 0.9871 |
| CXCL1 | 13.67 (13.67 / 48.02) | 13.67 (13.67 / 43.12) | >0.9999 |

*Mann-Whitney U-test
